# Supplementary material for: Population Genetic Structure Reveals Two Lineages of Amynthas triastriatus (Oligochaeta: Megascolecidae) in China, with Notes on a New Subspecies of Amynthas triastriatus
Source: Int J Environ Res Public Health. 2020 Feb 27;17(5):1538. doi: 10.3390/ijerph17051538 (PMC7084275; doi:10.3390/ijerph17051538)
Supplement: Supplementary file 1 [file ijerph-17-01538-s001.pdf]

Table A1. The GenBank accession numbers of the specimens used in this study.

Gene Code	COI	COII	ATP8	ND6	12S rRNA	16S rRNA	ND1
CQ16	NC720163	NC720604	MK210068	MK209957	NC721486	NC721927	NC721045
FJ10	NC719749	NC720190	MK210077	MK209966	NC721072	NC721513	NC720631
FJ65	NC719758	NC720199	MK210114	MK210008	NC721081	NC721522	NC720640
GD56	NC719785	NC720226	MK210088	MK209981	NC721108	NC721549	NC720667
GX35	NC719830	NC720271	MK210100	MK209993	NC721153	NC721594	NC720712
GX172	MK225628						
GX189	MK225627						
GX194	NC719919	NC720360	MK210155	MK210038	NC721242	NC721683	NC720801
GX198	NC719921	NC720362	MK210153	MK210046	NC721244	NC721685	NC720803
GX210	MK225626						
GZ01	NC719945	NC720386	MK210061	MK209951	NC721268	NC721709	NC720827
GZ41	MK225625						
GZ44	MK225624						
GZ22	NC719952	NC720393	MK210084	MK209974	NC721275	NC721716	NC720834
HB06	NC720016	NC720457	MK210053	MK209973	NC721339	NC721780	NC720898
HN15	NC720031	NC720472	MK210070	MK209959	NC721354	NC721795	NC720913
HN43	NC720051	NC720492	MK210095	MK209988	NC721374	NC721815	NC720933
JX33	MK225617						
JX36	NC720078	NC720519	MK210099	MK209992	NC721401	NC721842	NC720960
JX40	MK225616						
SC18	KF179569	KF179584	MK210067	MK209956	KF179559	KF179547	KF179595
GZ135	MK225623						
GZ137	MK225622						
GZ142	MK209741	MK209860	MK210143	MK210043	MK202312	MK202344	MK209937
GZ151	MK225619						
GZ153	MK209740	MK209859	MK210157	MK210040	MK202311	MK202343	MK209936
GZ158	MK225618						
AH60	MK209758						
AH72	MK225633						
AH81	MK209757						
AH86	MK209755	MK209832	MK210130	MK210034	MK202264	MK202186	MK209906
AH91	MK225632						
AH94	MK225635						
ZJ52	MK209743	MK209796	MK210089	MK209982	MK202226	MK202259	MK209897
FJ68	NC719760	NC720201	MK210112	MK210006	NC721083	NC721524	NC720642
FJ09	NC719733	NC720174	MK210079	MK209968	NC721056	NC721497	NC720615
GD83	NC719794	NC720235	MK210133	MK210037	NC721117	NC721558	NC720676
GX63	NC719831						
GX200	NC719922	NC720363	MK210152	MK210050	NC721245	NC721686	NC720804
HN04	NC720026						
HN34	NC720045	NC720486	MK210102	MK209995	NC721368	NC721809	NC720927
JX06	NC720056	NC720497	MK210054	MK209944	NC721379	NC721820	NC720938
JX26	NC720071	NC720512	MK210109	MK210002	NC721394	NC721835	NC720953
JX50	MK225615						
JX60	MK209713						
JX62	MK225614						
JX89	MK225613						
SC15	NC720103	NC720544	MK210071	MK209960	NC721426	NC721867	NC720985
ZJ02	NC720152	NC720593	MK210059	MK209949	NC721475	NC721916	NC721034
GZ136	MK209717	MK209862	MK210144	MK210044	MK202314	MK202316	MK209939
GZ143	MK225621						
GZ144	MK225620						

Table A1. *Cont.*

AH50	MK209737	MK209798	MK210091	MK209984	MK202228	MK202232	MK209899
AH67	MK225635						
AH69	MK225634						
AH92	MK209754	MK209830	MK210128	MK210032	MK202262	MK202184	MK209905
AH100	MK209753	MK209826	MK210125	MK210029	MK202284	MK202179	MK209927
AH101	MK225630						
AH104	MK225629						
ZJ51	MK209744	MK209797	MK210090	MK209983	MK202227	MK202231	MK209898
ZJ83	MK209768	MK209833	MK210132	MK210036	MK202265	MK202187	MK209907
ZJ87	MK225612						
ZJ93	MK209766	MK209828	MK210127	MK210031	MK202260	MK202182	MK209903
ZJ98	MK209765	MK209827	MK210126	MK210030	MK202285	MK202181	MK209902
ZJ103	MK225611						
Aporrectodea trapezoides	MH845535	MH110575	MK210148	MK209975	MK106038	MK103055	MK098757
